# Using Genetic Data to Determine Origin for Out‐Migrating Smolt and Returning Adult Steelhead Trout (*Oncorhynchus mykiss*) in a Southeast Alaska Drainage

**DOI:** 10.1002/ece3.70472

**Published:** 2024-10-25

**Authors:** Evan J. Barfuss, Bridey E. Brown, Shriya Sachdeva, Asher B. Smith, Frank P. Thrower, Charles D. Waters, Krista M. Nichols, Matthew C. Hale

**Affiliations:** ^1^ Department of Biology Texas Christian University Fort Worth Texas USA; ^2^ Auke Bay Laboratories Alaska Fisheries Science Center, National Marine Fisheries Service, National Oceanic and Atmospheric Administration Juneau Alaska USA; ^3^ Conservation Biology Division Northwest Fisheries Science Center, National Marine Fisheries Service, National Oceanic and Atmospheric Administration Seattle Washington USA

**Keywords:** conservation, DMAS‐qPCR, rainbow trout, smoltification, steelhead trout

## Abstract

*Oncorhynchus mykiss* is a partially migratory salmonid species, and many migratory populations (known as steelhead) have declined in recent decades in the western United States and Canada. Closely related resident populations (known as rainbow trout) may be an effective resource in the recovery of these declining migratory populations. However, the extent to which different populations of resident rainbow trout produce migratory individuals and how likely these individuals are to return as adults to spawn remains unknown. One limitation to answering these questions is the identification of loci that accurately segregate between migratory and resident populations. To address this limitation, we used existing genomic data from a well‐studied population of *O. mykiss* from Southeastern Alaska (Sashin Creek) to identify loci that segregate between phenotypes. We then utilized Double Mismatch Allele‐Specific qPCR (DMAS‐qPCR) to genotype 233 smolts out‐migrating from Sashin Creek and 99 returning adult steelhead trout across a five‐year period to determine (a) the origin of out‐migrating smolts and returning adults and (b) to quantify the extent to which the resident population contributes to the migratory population. Our results show that 37.3% of out‐migrating smolts were produced from resident parents, whereas 19.3% of returning adults had resident parents. Ultimately, these results demonstrate that resident populations of rainbow trout produce migrant offspring that successfully complete their migration and return to spawn, increasing population sizes and likely improving genetic diversity. Therefore, conservation efforts should consider landlocked resident populations for producing smolts when developing recovery plans for migratory steelhead populations.

## Graphical abstract

We find evidence that resident populations of rainbow trout can and do produce migratory smolts that can successfully return to spawn. There are statistically significant differences in return timing between migrants with migratory parents and migrants with resident parents suggesting there may be selection operating on return timing.

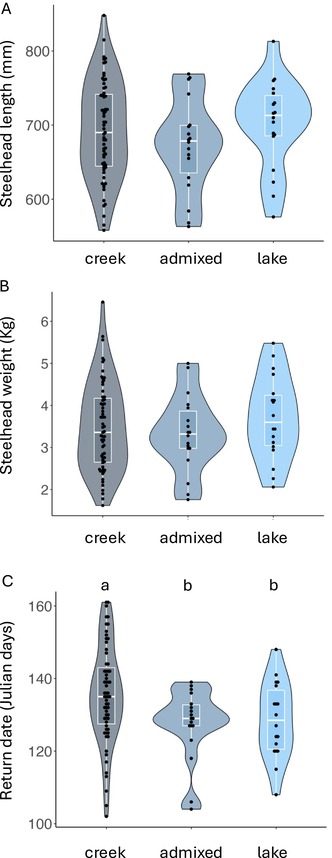

## 1 Introduction

Although the effects of climate change and human‐mediated habitat modification (i.e., building dams, reservoirs, deforestation, and habitat fragmentation) affect many taxa, migratory and partially migratory species are particularly at risk as migrants occupy different ecosystems during their life cycle (Wilcove and Wikelski 2008; Liedvogel, Akesson, and Bensch 2011). Such species often show plasticity in their behavior, with some individuals staying resident while others migrate to take advantage of seasonal resources (e.g., Dingle 2006; Chapman et al. 2012). For example, many populations of salmonids (salmon, trout, and charr) exhibit partial migration where some individuals within a population undergo smoltification (the process of changing from a freshwater‐adapted parr into a saltwater‐adapted smolt) and migrate, whereas others remain resident. This behavioral dichotomy involves life‐history trade‐offs in both survival and size and age at first reproduction (Pavlov and Savvaitova 2008; Sloat et al. 2014; Kendall et al. 2015). Residents typically have a higher chance of survival, become sexually mature at a younger age, and are not required to go through the energetic costs associated with smoltification, while migrants delay maturation but obtain a greater size and are more fecund (e.g., Thorpe 1994). Many factors can influence these trade‐offs and can result in year‐to‐year as well between population variation in which life history predominates (Stearns 1989; Fleming 1996; Kendall et al. 2015).

*Oncorhynchus mykiss* is an exemplary taxon to study life history development as many natural populations exhibit both ecotypes (i.e., migratory steelhead trout and resident rainbow trout). Although the exact mechanisms underlying the resident and migratory ecotypes are not completely understood, there is clear evidence that an individual's life history is influenced by both additive genetic effects (Thrower, Hard, and Joyce 2004; Nichols et al. 2008; Hale et al. 2013; Hecht et al. 2014) and environmental conditions such as body size, water temperature, lipid content, and photoperiod (Sloat et al. 2014). However, as alluded to above, it is important to note that life history development in rainbow trout is nuanced and can vary within and between populations.

Many migratory salmonid populations are decreasing at rates not mirrored in resident populations due to the utilization of different habitats and resources, and the increased energetic demands of migration. These declines have led to the designation of many populations of steelhead trout to be classified as either threatened or endangered under the Endangered Species Act (Busby et al. 1996; Waples 1991) in the United States. Conservation efforts such as habitat restoration and hatchery supplementation are in place to maintain and support steelhead populations. However, success of these programs has been limited, in part, due to changing ocean conditions (e.g., Cavole et al. 2016), a lack of purifying selection (Lynch and O'Hely 2001), inbreeding depression (Wang, Hard, and Utter 2002), and the potential for domestication selection and reduced relative reproductive success of hatchery‐reared fish (e.g., Ford 2002; Christie, Ford, and Blouin 2014). Moreover, the use of hatchery fish to supplement wild populations has the potential to integrate alleles associated with maladaptive phenotypes such as lower marine survival and changes to run timing (Christie, Ford, and Blouin 2014; Koch and Narum 2021; Thériault et al. 2011; Ford et al. 2006). Therefore, there is a need to evaluate additional strategies to aid in the recovery of migratory populations.

Previous studies suggest the genetic basis for anadromy in *O. mykiss* is polygenic and population‐specific with alleles associated with life history development not shared between different populations (e.g., Nichols et al. 2008; Hecht et al. 2013; Weinstein et al. 2019; Clare et al. 2023, but see Pearse et al. 2019 for an example of a large chromosomal inversion that is associated with life history development and is shared between different populations). Despite this, resident populations that reproductively isolated above barriers can retain genetic variance associated with anadromy (Pearse et al. 2014; Leitwein, Garza, and Pearse 2017; Campbell et al. 2021; Hale et al. 2013). Moreover, some of these populations have returned to anadromous behavior after removal of man‐made barriers (Fraik et al. 2021). Therefore, it is possible that the genetic basis for migration can be retained in residents despite seemingly strong selection against doing so. However, it is not known if, and to what extent, (a) resident populations produce anadromous offspring and (b) these offspring successfully return to reproduce.

Here, we investigated a population of *O. mykiss* from Sashin Creek in Southeast Alaska, where the genetic basis for life history determination has been well studied (Thrower, Hard, and Joyce 2004; Hecht et al. 2012; Hale et al. 2013). The main goal of this study was to utilize DMAS‐qPCR to genotype out‐migrating smolts and returning adult steelhead at multiple loci that appear to be associated (most likely via genetic drift) with origin. We used these assays to test and categorize smolts and returning adults as either originating from the lake (i.e., resident parents) or from the creek (i.e., migratory parents). These data allowed us to answer if, and to what extent, resident parents produce migratory offspring and if these offspring are as likely to return to spawn as those produced by migratory parents. A second goal of this study was to investigate if the proportion of lake‐produced smolts was influenced by environmental factors. Our results provide a deeper understanding of the contribution of lake produced smolts to yearly outmigration and help determine specific factors that may influence variation in smolt origin. We hope that the approaches used in this study could then be applied to other populations to aid in the development of population‐specific *O. mykiss* recovery plans.

## 2 Materials and Methods

### 2.1 Study Site and Sample Collection

Samples for this study were collected from Sashin Creek at the National Oceanic and Atmospheric Administration's Little Port Walter Research Station, located on southeast Baranof Island in Alaska, USA. The Sashin system, which has been extensively studied in previous anadromy research, contains two populations of *O. mykiss*—Sashin Creek is open to the Pacific Ocean and contains a mostly anadromous population, while Sashin Lake contains a resident population and is separated from Sashin Creek by two barrier waterfalls (Thrower et al. 2004). The population in Sashin Lake was established in 1926 by a transplant of ~80 trout from Sashin Creek. Fish and tissue samples analyzed in this study were obtained from 2015 to 2021 at a weir operated at the mouth of Sashin Creek, from both out‐migrating steelhead smolts and returning adult steelhead. Fish were weighed (grams for smolts, kilograms for adults), measured (fork length; mm), and the Julian day of either out‐migration (smolts) or return (adults) recorded. Fin clips from both smolts and adults were collected from the left ventral, axillary process, or the caudal fin and placed in 95% ethanol for subsequent DNA extraction. Out‐migrating smolts were released downstream of the weir to enter the Pacific Ocean while returning adult steelhead were then moved upstream of the weir to complete their migration and to reproduce. DNA was extracted from fin clips using Qiagen DNeasy Blood & Tissue kits according to the manufacturer's protocol and subsequently standardized to a concentration of 50 ng/μL for genotyping. All DNA was checked for quality by running 3 μL on a 1.5% agarose gel stained with Gel Red (manufacturer?) and viewed under UV light.

### 2.2 Identification of Polymorphic Markers

Three sources of sequence data from previous studies of the Sashin Creek/Lake system were used to identify SNP markers that segregate between anadromous steelhead trout collected in Sashin Creek and mature rainbow trout collected in Sashin Lake: (1) RNA‐seq data generated from 2‐year‐old smolts and residents initiated as part of a common‐garden study focused on the genetic basis of migration (Hale et al. 2016), (2) pooled‐sequencing data from returning adult steelhead and mature rainbow trout from the lake (Clare et al. 2023), and (3) low‐coverage whole‐genome sequence data from 36 mature steelhead and rainbow trout collected in 2015 and 2017. The methods utilized to identify SNPs that segregate between the two ecotypes varied depending on the dataset and will be discussed below. For all three methods, loci in genomic locations under tetrasomic inheritance (Campbell et al. 2019) were removed from consideration. Additionally, multiple loci found within 1 kb of each other were treated as a single locus to avoid selecting genetic markers in high linkage disequilibrium.

### 2.3 RNA‐Seq Data

RNA‐seq data from whole brain RNA‐extractions from 23 fish (12 from an anadromous family cross (A × A) and 11 from a resident rainbow family cross (R × R)) were used (sampling and sequencing details are provided in Hale et al. 2016). The A × A samples had all undergone smoltification (~2‐year‐old fish) and included both sexes. The R × R samples showed no signs of undergoing smoltification, as the males had reached sexual maturity, and the females had a condition factor > 1 (Hecht et al. 2012). Quality filtering was performed using Trimmomatic v0.32 (Bolger, Lohse, and Usadel 2014) with default parameters to remove sequencing adapters and low‐quality bases (Q‐values < 20). Quality‐filtered reads were subsequently aligned to the *O. mykiss* genome (Omyk_1.0; GCA_002163495.1) with the program *STAR* v2.7.3 using default parameters (Dobin et al. 2013). Alignments were converted into BAM files using the mpileup function in the program *Samtools* v1.19 (Danecek et al. 2021), with a minimum quality score of 30 for both nucleotide calling and genome alignment. SNPs were scored using *ANGSD* v0.930 (Korneliussen, Albrechtsen, and Nielsen 2014) with parameters set to a minimum minor allele frequency of 0.10, maximum minor allele frequency of 0.45 (to reduce the likelihood of paralogous sequence alignments), and cutoff SNP p‐value of 1e‐5. Statistical associations between origin (i.e., A × A vs. R × R) and SNPs were determined using *PLINK v1.9* (Chang et al. 2015) using a Fisher's exact test with a Bonferroni corrected *p*‐value for statistical significance. The top 30 SNPs with the strongest association (i.e., smallest *p*‐value) between A × A and R × R families were selected for further investigation.

### 2.4 Pooled‐Seq Data

The second group of loci were selected based on pooled‐sequencing data generated by Clare et al. (2023) comparing a pool of steelhead to a pool of resident individuals. The pools used for sequencing came from 40 returning adult steelhead from Sashin Creek and 40 resident rainbow trout from Sashin Lake, equally sampled between males and females. For this study, we focused on SNPs that exhibited a fixed F_ST_ difference (i.e., *F*_ST_ = 1) between the two pools as these loci are most likely to represent fixed (or nearly so) allelic differences between resident individuals from Sashin Lake and steelhead from Sashin Creek.

### 2.5 Whole‐Genome Sequencing SNPs

Low‐coverage whole‐genome sequencing was used to locate SNPs segregating between resident rainbow trout and migratory steelhead. Briefly, DNA was extracted from fin clips from 36 individuals (18 resident fish from Sashin Lake and 18 migratory fish from Sashin Creek) using Qiagen's DNeasy kit according to manufacturer's protocol. Samples were chosen based on phenotype (i.e., returning adult steelhead or sexually mature residents), and an equal number of individuals from each sex were sampled from each phenotype (nine of each sex). Genomic DNA was sent to NovoGene for low‐coverage whole‐genome paired‐end 150‐bp sequencing. Sequencing was performed on an Illumina NovaSeq S4 using TruSeq library prep. Sequences were quality filtered using Trimmomatic to remove Illumina adapter sequences and low‐quality base pairs (*Q* values < 20). Quality‐filtered reads were then aligned to V2 of the rainbow trout genome (GenBank assembly accession GCA_002163495.1; Pearse et al. 2019) using BWA‐mem with default parameters. Aligned reads were then transformed into sorted BAM files for SNP discovery using *Samtools* v0.1.19 (Li and Durbin 2009). Sorted BAM files were then analyzed for SNPs using *ANGSD* v0.918 (Korneliussen, Albrechtsen, and Nielsen 2014) with the following parameters: ‐minMaf = 0.05, minInd = 18, minMapQ 20, minQ 20, SNP_SNP‐*p*val 1E‐6. Analyses of resulting SNPs were performed using *PLINK v1.9*, and SNPs with *F*_ST_ > 0.5 were further investigated.

### 2.6 Confirmation of SNPs via Sanger Sequencing

Although next generation sequencing is an excellent tool for uncovering genome‐wide variation, we used Sanger Sequencing to sequence 29 loci to verify the presence of SNPs that segregated between resident lake and anadromous creek populations. Primers were designed using either Primer3Web or the NCBI's Primer BLAST function with a median annealing temperature of 60°C and a median length of 20 nucleotides (see File S1 for primer sequences). Samples were amplified on a Bio‐RadT100 Thermal Cycler using QuantaBio AccuStart II PCR SuperMix. The standard PCR protocol was a denaturing temperature of 95°C, an annealing temperature of 60°C, and an elongation temperature of 72°C for 35 cycles. Samples were cleaned using Exo‐Sap (NEB) followed by a sequencing reaction using Big Dye v3.1 chemistry (ABI). The resulting samples were analyzed on an Applied Biosystems 3130xl Genetic Analyzer. To confirm SNPs, chromatographs were imported into Sequencher v5.4.6 (Gene Codes) and aligned into contigs, with chromatographs displaying a quality score below 70% excluded from analysis. At nucleotide sites where Sequencher identified a lack of consensus, chromatographs were visually inspected to determine whether the report was due to a sequencing artifact or due to the presence of differing alleles between sequences. Once a minimum of 10 samples from each population had been genotyped, a Freeman–Halton extension of Fisher's exact test was used to test for significant differentiation in allele frequencies between lake and creek populations. Loci that exhibited a *p*‐value of 0.05 or less were recorded for subsequent use in genotyping. Allele frequencies were also tested for deviations from Hardy–Weinberg Equilibrium (within life histories) via a Chi‐squared test to confirm whether any loci were under selective pressure. Any locus that was not in Hardy–Weinberg Equilibrium was removed from further consideration.

### 2.7 Development of DMAS‐qPCR Primers and Execution of Genotyping Assays

To develop a suite of putatively neutral loci that can be routinely used to discriminate between life history types, 233 out‐migrating smolts and 99 returning adult steelhead were genotyped at eight segregating SNP loci using double‐mismatch allele‐specific qPCR (DMAS‐qPCR) assays. Briefly, forward primers were designed so that the 3′ end precisely overlapped the target SNP, with distinct forward primers designed for each possible SNP allele (i.e., one forward primer contained the “anadromous creek” allele and the other forward primer the “resident lake” allele; hereafter referred to as the “creek allele” and the “lake allele”, respectively; primer details are given in File S1). Forward primers were designed with an intentional allelic mismatch three nucleotides upstream of the SNP as described by Lefever et al. (2019). Primer sets used a common reverse primer and had target amplicon lengths between 40 and 80 base pairs. Samples were genotyped by performing separate qPCR assays with both versions of the forward primer. qPCR of samples was performed on an Applied Biosystems StepOnePlus Real‐Time PCR System. qPCR assay mixtures were made with a DNA concentration of 5 ng/μL, primer concentration of 0.5 ng/μL, and total volume of 10 μL. Assays were performed in triplicate and the *C*q (the point at which the PCR crossed a locus specific threshold) values averaged. Any score with a standard deviation > 0.5 were removed. Genotype calls were made by comparing the Cq scores of samples assayed with the lake and creek forward primers for each sample, and these scores were used to classify genotypes as either homozygous (i.e., for the lake allele or the creek allele) or heterozygous.

To validate that the assays were working as intended, 10 adult samples (five resident rainbow trout from Sashin Lake and five migratory steelhead from Sashin Creek) previously genotyped via Sanger sequencing were re‐genotyped at the final suite of putatively neutral markers—eight loci—using the DMAS‐qPCR assays. For each locus, we tested to see whether (a) samples were consistently amplifying in both creek and lake primers, (b) the comparison of creek and lake Cqs resulted in clear grouping, and (c) the genotypes determined by the assay corroborated genotypes from Sanger sequencing. If the assay for a locus failed to meet those conditions, the primers were re‐designed if possible, or the locus was removed from analysis.

### 2.8 Differentiation of Anadromous Creek and Resident Lake Samples

Sample genotypes were categorized as being either homozygous for the creek allele, homozygous for the lake allele, or heterozygous (i.e., one of each of the alleles) for all eight loci. These genotypes were then used to categorize a sample as being from the creek, the lake, or admixed (i.e., alleles suggesting origin from both populations). Admixed samples were those which had fewer than six loci (i.e., < 75%) in agreement with respect to origin. Lastly, all individuals were sex‐typed using the *Omy_Y1* locus following protocols outlined in Brunelli et al. (2008).

### 2.9 Analyses of Phenotypic Data

Quantitative data were recorded for both out‐migrating smolts and returning adult steelhead from samples caught at the Sashin Creek weir. Associations between fork length, weight, either out‐migration date (smolts) or return date (adults), and sample origin (i.e., creek, admixed, or lake) were investigated for all genotyped samples using two‐way ANOVAs followed by a post hoc Tukey test. For each test, year—together with the interaction between year and origin—was included as a co‐factor. All analyses were conducted in R (R Core Team 2022; aov and tukeyHSD commands) with alpha set to 0.05.

### 2.10 Associations Between Lake Origin Smolts and Environmental Variables

We investigated whether several key environmental variables—namely, freshwater temperature, total rainfall, and total snowfall at Sashin Creek—were associated with the proportion of out‐migrating lake‐produced smolts. Specifically, we tested for associations between environmental conditions in the fall, winter, and spring prior to outmigration (smolts typically out migrate from May to July about 2 years after hatching; McCormick 2013). For freshwater temperature, associations were tested between the proportion of lake‐produced smolts each year and average monthly water temperature for each of the 9 months (Sept. to May) prior to mean outmigration date. For example, average monthly freshwater temperatures between September 2015 and May 2016 were compared to the proportion of lake‐produced smolts that out migrated in 2016. Associations with rainfall were tested in the same manner using total monthly rainfall (in mm). Associations were tested using a regression analysis with significance determined via Pearson's correlation coefficient. Associations between snowfall and the proportion of lake‐produced smolts were performed in a similar way; we used total snowfall between September 1st and April 30th and tested for associations with the proportion of lake‐produced smolts using a two‐tailed paired t‐test that assumed unequal variance. All three analyses were performed in base *R* with alpha set to 0.05.

## 3 Results and Discussion

A total of 105 candidate SNPs were identified (30 from RNA‐seq data set, 39 from pool‐seq data, and 36 from lcWGS data), of which 69 were removed due to them being in high linkage disequilibrium with multiple loci or because they were in known tetrasomically inherited regions. Of the remaining 36 loci, 14 were found to accurately segregate between resident rainbow trout from Sashin Lake and anadromous steelhead from Sashin Creek based on Sanger sequencing and were used for primer design. Two of these loci failed to produce usable primers, and four loci were removed due to inconsistent separation of genotypes. The remaining eight loci all demonstrated consistent amplification and a high degree of separation between creek and lake samples. No locus was out of Hardy–Weinberg Equilibrium (i.e., were putatively neutral) and all loci produced a significant Freeman–Halton extension of the Fisher's exact test confirming that those loci showed significant differences in allele frequency between origin (*p* < 0.001).

**FIGURE 1:**
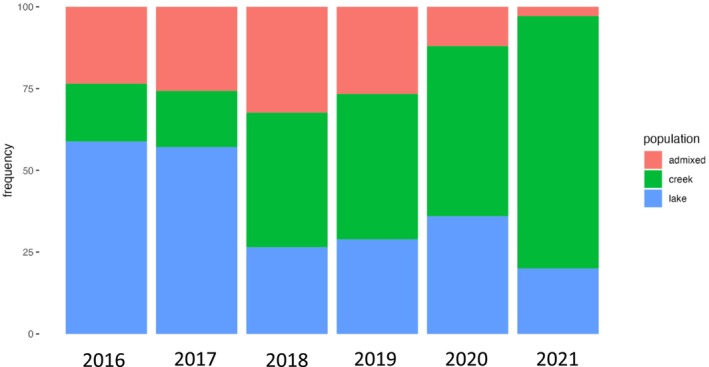
Stacked bar plot showing the proportions of out‐migrating smolts that are Sashin Creek, admixed, or Sashin Lake in origin.

**FIGURE 2:**
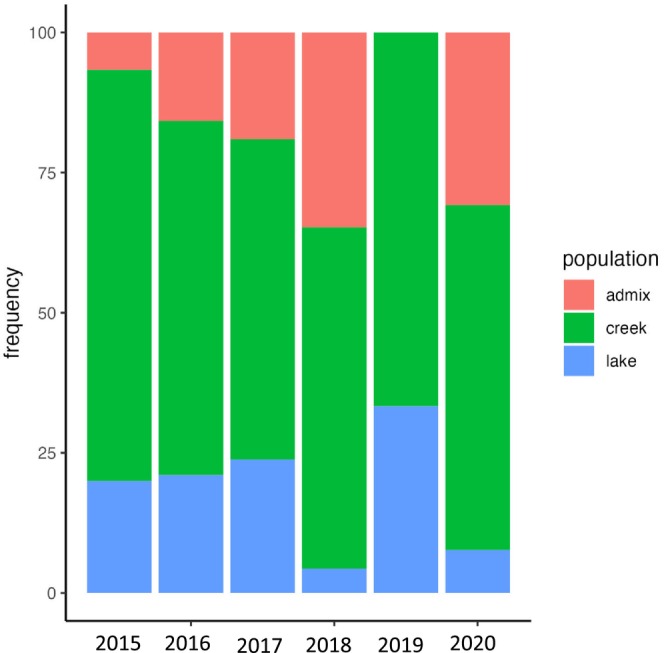
Stacked bar plot showing the proportions of returning adult steelhead that are Sashin Creek, admixed, or Sashin Lake in origin.

Eight loci were genotyped in 233 out‐migrating smolts (collected from 2017 until 2021) and 99 returning adult steelhead (2015 to 2020). All three origins (i.e., Sashin Lake, admixed, and Sashin Creek) were observed for each year for both out‐migrating smolts and returning adult steelhead except for the 2019 adult steelhead that did not have any admixed samples. There was a significant difference in the proportions of out‐migrating smolts that originated from Sashin Lake, Sashin Creek, or were admixed for four of 6 years and for all years for returning adult steelhead (Figures 1 and 2; Chi‐squared test; *p* < 0.001). For the out‐migrating smolts, there was a significant bias towards lake production in 2016 and 2017 (58.8% and 57.1%, respectively) and a bias towards creek production in 2020 and 2021 (52% and 77.1%, respectively, although note that weir operations only spanned ~60% of the smolt migration timing in 2021 due to personnel limitations). No statistically significant differences with origin were found for smolts leaving Sashin Creek in 2018 and 2019 (Figure 1). These results suggest not only that Sashin Lake produces out‐migrating smolts but also that the genetic origin of smolts leaving the Sashin drainage varies from year to year. Presumably, this is because environmental conditions vary both between the creek and the lake and between years. Indeed, we found that average freshwater temperature in February was significantly and positively associated with the proportion of out‐migrating smolts of lake origin (*R*_2_ = 0.58; *F* = 5.522, *p* < 0.05). We hypothesize that warmer freshwater temperatures increase food availability and growth potential in Sashin Lake and therefore lead to higher smolt development. We also found significant associations between rainfall amount and the proportion of lake‐origin smolts for every month from December until March, with higher rainfall being associated with an increase in the proportion of lake origin smolts (e.g., December rainfall totals: *R*_2_ = 0.677; *F* = 8.388, *p* < 0.01). However, we did not find an association between total snowfall and the proportion of lake‐produced smolts (*t* = 1.725, *p* = 0.128), suggesting that total snowfall is not a predictor of the proportion of smolts leaving Sashin Lake. Other studies have also found associations between smoltification and water temperature, flow rate, and food availability (Benjamin et al. 2013; Berejikian, Campbell, and Moore 2013; Sloat and Reeves 2013). However, the relative importance of these variables likely varies both temporally and between populations, and further research is required to fully understand how such factors are associated with smoltification.

The composition of out‐migrating smolts contrasted with the origins of returning adult steelhead, which were significantly creek biased (Figure 2: *p* < 0.001) for all years, suggesting that returning adult steelhead are more likely to be produced from creek parents than lake parents. Previous common‐garden experiments investigating the influence of parentage on marine survival in the Sashin Creek system found the same pattern, confirming that smolts produced by anadromous parents are more likely to return to spawn than smolts produced by resident parents (Thrower and Joyce 2004; Thrower et al. 2008; Thrower and Hard 2009). Although the reproductive success of the adults produced by resident parents has not been quantified, our finding indicates that barriers to gene flow for ~100 years does not stop smolt production (Thrower et al. 2004) and has implications regarding utilizing resident populations as sources to produce migratory smolts for population recovery (Thrower et al. 2008).

One interesting component of our dataset is the identification of smolts and adults with admixed parentage. We interpret these samples as having mixed parentage or grand parentage, presumably due to either resident rainbow trout moving downstream from Sashin Lake and reproducing with returning adult steelhead in Sashin Creek, or lake produced smolts successfully returning to Sashin Creek and breeding with creek produced steelhead. However, gene flow between populations appears to be rare due to (a) the identification of loci that segregate with respect to origin and (b) the relatively low proportion of admixed returning adult steelhead. Although it is known that Sashin Creek contains a resident population, we suspect either that the number of sexually reproducing resident individuals in Sashin Creek is low or that there are barriers to gene flow between ecotypes. Such barriers could be selective mating or (more likely) reduced likelihood of admixed smolts returning to spawn. We also performed sex‐typing assays on both out‐migrating smolts and returning adults. These data suggest that there is substantial female sex‐bias both in out‐migrating smolts (Table 1: *t* = 8.181, *p* < 0.0001) and returning adults (Table 2: *t* = 3.212, *p* = 0.024), confirming previous studies that suggest females obtain greater benefit from migrating than males (Ohms et al. 2013).

**TABLE 1:** Data showing origins of out‐migrating smolts. Each sample was genotyped using the eight diagnostic loci and sex‐typed (apart from 2019 smolts) using the OmyY1 locus. Samples were classified by origin based on their alleles. At least six loci had to show concordance for classification as lake or creek origin. Loci with fewer than six loci showing concordance were classified as admixed as such samples suggest mixed parentage.

Year	*n*	Sex	Total	Lake	Admix	Creek
2016 smolts	34	M	9	4	2	3
		F	25	16	6	3
2017 smolts	35	M	6	2	1	3
		F	29	18	8	3
2018 smolts	34	M	6	1	2	3
		F	28	8	9	11
2019 smolts	45	X	X	13	12	20
2020 smolts	50	M	11	0	3	8
		F	39	18	3	18
2021 smolts	35	M	7	1	0	6
		F	26	6	1	19
		X	2	0	0	2
Total	233			87	47	99

**TABLE 2:** Data showing origins of returning adult steelhead. Each sample was genotyped using the same eight loci as above and sex‐typed using the OmyY1 locus. Samples were classified by origin based on their alleles. At least six loci had to show concordance for classification as lake or creek origin. Loci with fewer than six loci showing concordance were classified as admixed.

Year	*n*	Sex	Total	Lake	Admix	Creek
2015	12	M	7	1	1	4
		F	8	2	0	4
2016	19	M	4	0	1	3
		F	15	4	2	9
2017	20	M	6	0	2	4
		F	14	5	1	8
2018	23	M	2	0	3	3
		F	6	1	5	11
2019	12	M	3	0	0	3
		F	9	4	0	5
2020	13	M	3	0	2	1
		F	10	1	2	7
Total	99			17	13	58

### 3.1 Analyses of Phenotypic Data Associated With Origin

**FIGURE 3:**
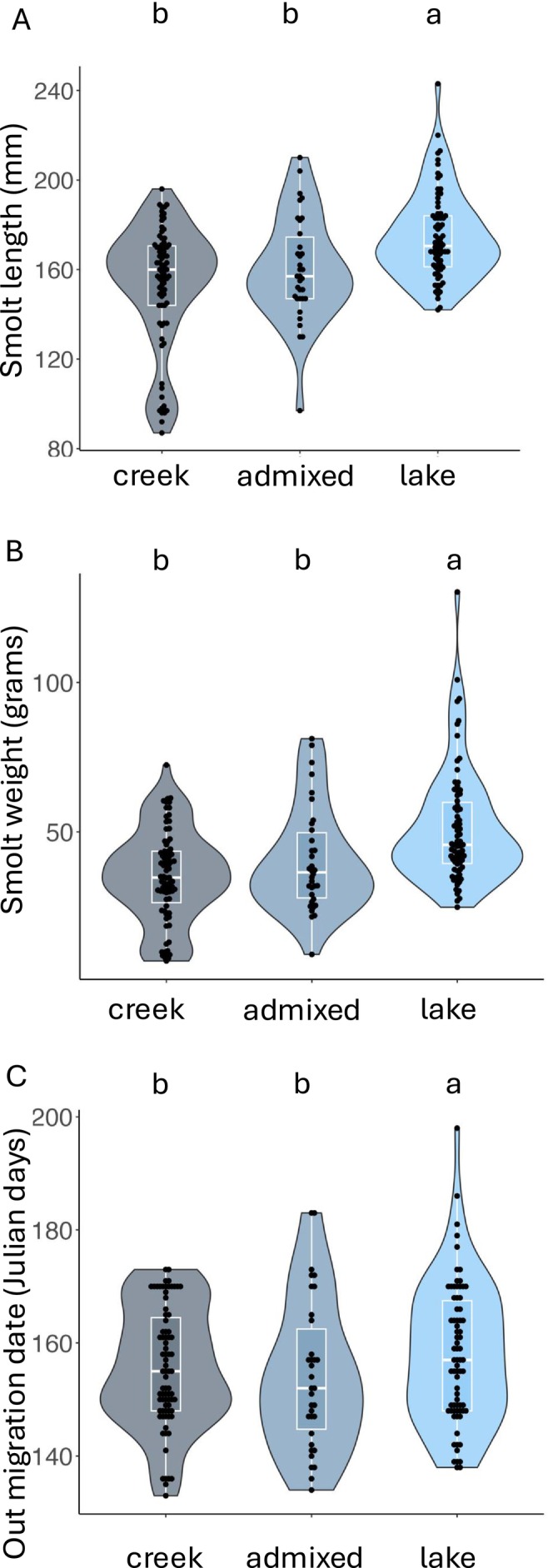
Violin and box plots showing data distribution for fork length (A), weight (B), and out‐migration date (C) for genotyped smolts. All three phenotypes were significantly associated with genetic origin (creek, admixed, or lake) when analyzed with a two‐way ANOVA with year categorized as a co‐factor. Results from post hoc Tukey tests are also shown.

We analyzed phenotypic data associated with genotyped smolts and adult steelhead to investigate if there were significant differences in weight, length, and either out‐migrating date (smolt) or return date (adults) between different origins (i.e., lake produced, creek produced, or admixed). We found phenotypic differences in fork length and weight in out‐migrating smolts, suggesting samples from the lake are larger than admixed or creek produced smolts (length: *F* = 30.024, *p* < 0.0001; weight: *F* = 32.437, *p* < 0.0001; Table 3: Figure 3A,B). These differences could be due to variation in environmental factors between habitats such as food availability and growth potential. Alternatively, it could be due to a difference in age at outmigration if lake‐produced smolts are, on average, older than creek‐produced smolts, but this has yet to be investigated using scale samples. The common garden experiments mentioned earlier (Thrower and Joyce 2004; Thrower et al. 2008; Thrower and Hard 2009) did not find significant differences in length and weight in smolts produced by lake residents compared to anadromous steelhead, further suggesting this size difference is due to environmental factors and not additive genetic effects. Neither year nor the interactions between year and either metric, were statistically significant. Out‐migration date was significantly associated with origin (*F* = 4.062, *p* = 0.021; Table 3: Figure 3C), with smolts produced from the lake leaving on average 5 days later than smolts of creek origin. This most likely is due to a combination of environmental factors, including the lake likely being cooler than the creek (reduced development rate) and the increased distance between the lake and the weir (~3 km). It was somewhat surprising that there was no interannual variability in out‐migration date, as environmental factors such as water temperature and stream flow can affect life history development and migration timing (e.g., Berejikian, Campbell, and Moore 2013; Doctor et al. 2014); no interaction was found between origin and year (*F* = 0.537, *p* = 0.844; Table 3). However, it is important to emphasize that the data used in this study comes from a six‐year period and follow‐up studies that investigate a larger longitudinal data set could very well find variance in out‐migration date.

**TABLE 3:** Table of two‐way ANOVAs testing for interactions between origin (i.e., creek, admixed, or lake) with year of outmigration for migratory smolts leaving Sashin Creek.

Dependent variable	Origin	Year	Year × Origin
Fork length	*F* = 30.024, *p* < 0.0001	*F* = 1.625, *p* = 0.204	*F* = 0.532, *p* = 0.467
Weight	*F* = 32.437, *p* < 0.0001	*F* = 1.874, *p* = 0.173	*F* = 0.078, *p* = 0.781
Julian days	*F* = 4.062, *p* = 0.021	*F* = 0.775, *p* = 0.570	*F* = 0.537, *p* = 0.844

**FIGURE 4:**
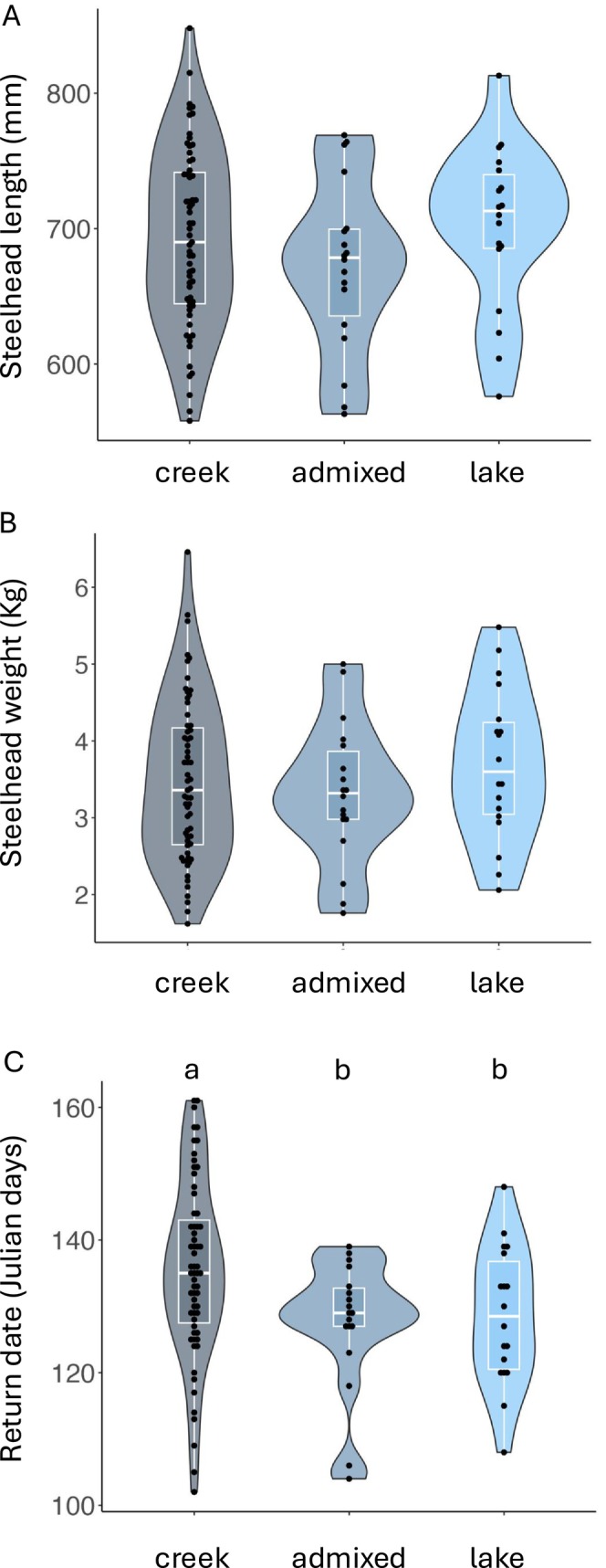
Violin and box plots showing data distribution for fork length (A), weight (B), and return date (C) for genotyped adult steelhead. Only return date was significantly associated with genetic origin (creek, admixed, or lake) when analyzed with a two‐way ANOVA with year categorized as a co‐factor. Results from post hoc Tukey tests are also shown.

No associations between origin and fork length and weight were found for returning adults (*F* = 1.127, *p* = 0.329 and *F* = 0.732, *p* = 0.484, respectively; Table 4, Figure 4A,B). These data suggest that origin has no effect on adult body mass, and similar results were reported in steelhead from controlled crosses (Thrower et al. 2008). However, return date were significantly associated with origin (*F* = 8.187, *p* = 0.006; Table 4, Figure 4C), with adults that originated from the creek returning on average 15 days later than adults that originated from Sashin Lake, and this phenotypic difference was consistent across years. We do not yet understand the adaptive significance of this difference or its influence on reproductive success (i.e., fitness). Several studies from multiple species of salmonids have found additive genetic effects associated with return date (Smoker, Gharrett, and Joyce 1994; Neira et al. 2006; Narum et al. 2024), suggesting that the difference in return time between the lake and creek adults may have a genetic basis. Importantly, return timing and spawn timing are known to exhibit strong phenotypic and genetic correlations in some salmonid populations (e.g., Quinn, Unwin, and Kinnison 2000), with potential implications for fitness. Later spawn timing in salmonids has been associated with higher fitness due to a reduced likelihood of redd disturbance by returning conspecifics (Hendry et al. 2004) and reduced predation (Brännäs, 1995). Alternatively, later spawn timing may negatively affect fitness because individuals face increased competition for mates and territories (Foote 1990), and their offspring may suffer higher mortality after emergence due to increased predation from older juveniles (Einum and Fleming 2000). However, the strength and direction of the associations between arrival date on the spawning grounds and fitness likely varies between studies, species, systems, and years (Anderson et al. 2010; Kodama, Hard, and Naish 2012; Sard et al. 2015). Additional analyses, such as pedigree reconstruction, will be needed to understand the effects of the 15‐day difference in return timing on fitness in the Sashin Creek system.

**TABLE 4:** Table of two‐way ANOVAs testing for interactions between origin (i.e., creek, admixed, or lake) with year of returning for migratory adult steelhead sampled at Sashin weir.

Dependent variable	Origin	Year	Year × Origin
Fork length	*F* = 1.127, *p* = 0.329	*F* = 3.643, *p* = 0.005	*F* = 0.835, *p* = 0.586
Weight	*F* = 0.732, *p* = 0.484	*F* = 4.708, *p* < 0.0001	*F* = 0.693, *p* = 0.713
Julian days	*F* = 8.187, *p* = 0.006	*F* = 0.288, *p* = 0.594	*F* = 0.788, *p* = 0.378

### 3.2 Future Directions for Sustainability of Migrant *O. mykiss* Populations

*Oncorhynchus mykiss* is one of the most widespread and well‐studied anadromous fish species, with considerable interest in rebuilding declining anadromous populations. One way to address this challenge would be to identify alleles that accurately allow the life history development of an individual to be predicted. Anadromous and resident individuals from the same population are more genetically similar to each other than they are to fish of the same ecotype from a different population (Narum et al. 2004; Thrower et al. 2008; Leitwein, Garza, and Pearse 2017). Thus, it would be ideal for recovery programs to focus on utilizing fish from the same system rather than releasing smolts from other wild or hatchery populations, and such an opportunity exists in sequestered or land‐locked resident populations. The data presented herein come from such a population and demonstrate that sequestered resident populations produce smolts that successfully return to spawn. However, the interplay between genetics and the environment mean that genotyping methods will likely necessitate the development of population‐specific assays. Although genotyped loci may be population‐specific, it will allow for a more nuanced and specific understanding of (a) if land‐locked resident populations produce out‐migrating smolts, (b) if those smolts are as likely to return to spawn as smolts produced by anadromous parents, and (c) if those smolts are as likely to contribute to the gene pool as smolts produced by anadromous parents. These data could increase our understanding of the genetic basis of migration and help preserve migratory populations.

## Author Contributions

**Evan J. Barfuss:** formal analysis (equal), investigation (equal), methodology (lead), visualization (equal), writing – original draft (lead). **Bridey E. Brown:** formal analysis (supporting), investigation (supporting), supervision (equal), validation (equal), visualization (supporting), writing – review and editing (supporting). **Shriya Sachdeva:** formal analysis (supporting), methodology (supporting), visualization (supporting). **Asher B. Smith:** formal analysis (supporting), methodology (supporting), validation (supporting), visualization (supporting). **Frank P. Thrower:** conceptualization (equal), project administration (equal), writing – review and editing (supporting). **Charles D. Waters:** investigation (supporting), methodology (supporting), writing – original draft (supporting), writing – review and editing (supporting). **Krista M. Nichols:** conceptualization (equal), project administration (lead), supervision (equal), writing – review and editing (supporting). **Matthew C. Hale:** formal analysis (equal), funding acquisition (lead), project administration (equal), supervision (lead), writing – original draft (equal).

## Conflicts of Interest

The authors declare no conflicts of interest.

## Supporting information


**File S1.** Primer sequences of successful DMAS‐qPCR loci.

**File S2.** Genotype data from *O. mykiss* samples (smolts and returning steelhead) used the study.

**File S3.** Phenotypes and origin for smolts and returning steelhead. The phenotypes sampled are length (mm) weight (g for smolt, kg for steelhead) and either out migration date for leaving smolts or return date for returning steelhead.

**File S4.** Monthly rainfall and temperature data for Sashin Creek recorded from January 2015 until December 2021.

## Data Availability

Both genotype and phenotype data associated with out‐migrating smolts and returning steelhead are available in Files S2 and S3 respectively. We also uploaded environmental data to File S4 that were used in testing for associations with rainfall and temperature and the proportion of lake produced smolts.
