# Supplementary material for: Using Genetic Data to Determine Origin for Out‐Migrating Smolt and Returning Adult Steelhead Trout (Oncorhynchus mykiss) in a Southeast Alaska Drainage
Source: Ecol Evol. 2024 Oct 25;14(10):e70472. doi: 10.1002/ece3.70472 (PMC11502969; doi:10.1002/ece3.70472)
Supplement: Supplementary file 1 — File S1. Primer sequences of successful DMAS‐qPCR loci. [file ECE3-14-e70472-s004.docx]

Supp. File 1: Primer Sequences of successful DMAS-qPCR loci.

| Locus | Sequencing method | chromosome | Location of locus (MB) | SNP allele | Forward Primer | Reverse Primer | Annealing Temp |
| --- | --- | --- | --- | --- | --- | --- | --- |
| Chr1 | lcWGS | 1 | 22.12 | C/T | TGCTGTCAAAACAACTGTT**G**AC**[C/T]** | GACTCACATTGTTGTGCTAGGG | **59.5°C** |
| Gal-R1 | RNA-seq | 3 | 35.02 | C/G | CGCACAAATGCCTTCATA**G**GT**[C/G]** | TGCATTCAAGAGACTCAAGAGAC | **60°C** |
| PAAL | RNA-seq | 11 | 19.29 | G/T | GCAATATTAGGAGGGGTTGT | TATCAAGTTCCGTTTTTATT**G**TT**[C/A]** | **59°C** |
| GCOAD | RNA-seq | 12 | 70.62 | T/G | TCATGGAATACCCCAAATACA**G**AT**[T/G]** | GCAGTTTAAATAATCATGCCATTGC | **58.5°C** |
| Methyl-26 | RNA-seq | 13 | 12.94 | T/C | TTAAACTCACCTGGCTAAAGA**G**TG**[T/C]** | GGCAACATGTCACCCACTTC | **60°C** |
| Puromycin | RNA-seq | 13 | 17.91 | A/G | TTTGCAATAATTACACTGAAG**G**GG[**A/G**] | CAAGTGTGTGTAGATCGGTCA | **59°C** |
| TSC-22 | RNA-seq | 14 | 76.68 | A/T | GAGATGCTGGACTGTTGCTG | TGATGTTCTGAATCATGATTA**G**GG**[T/A]** | **58°C** |
| Chr20 | lcWGS | 20 | 5.55 | T/C | CCAAAGGCCAGCAGATAG**G**GA**[T/C]** | TTTATGTACAAACCAGCCCACAGG | **62°C** |
